# Evolution of the α-Subunit of Na/K-ATPase from *Paramecium* to *Homo sapiens*: Invariance of Transmembrane Helix Topology

**DOI:** 10.1007/s00239-016-9732-1

**Published:** 2016-03-10

**Authors:** Gene A. Morrill, Adele B. Kostellow, Lijun Liu, Raj K. Gupta, Amir Askari

**Affiliations:** Department of Physiology and Biophysics, Albert Einstein College of Medicine, Bronx, NY 10461 USA; Department of Biochemistry and Cancer Biology, University of Toledo Health Science Campus, Toledo, OH 43614 USA

**Keywords:** Na/K-ATPase α-subunit, Evolution, Protein domains, Transmembrane helix, Cell signaling, Helix–helix interactions

## Abstract

Na/K-ATPase is a key plasma membrane enzyme involved in cell signaling, volume regulation, and maintenance of electrochemical gradients. The α-subunit, central to these functions, belongs to a large family of P-type ATPases. Differences in transmembrane (TM) helix topology, sequence homology, helix–helix contacts, cell signaling, and protein domains of Na/K-ATPase α-subunit were compared in fungi (*Beauveria)*, unicellular organisms (*Paramecia*), primitive multicellular organisms (*Hydra*), and vertebrates (*Xenopus*, *Homo sapiens*), and correlated with evolution of physiological functions in the α-subunit. All α-subunits are of similar length, with groupings of four and six helices in the N- and C-terminal regions, respectively. Minimal homology was seen for protein domain patterns in *Paramecium* and *Hydra*, with high correlation between *Hydra* and vertebrates. *Paramecium* α-subunits display extensive disorder, with minimal helix contacts. Increases in helix contacts in *Hydra* approached vertebrates. Protein motifs known to be associated with membrane lipid rafts and cell signaling reveal significant positional shifts between *Paramecium* and *Hydra vulgaris,* indicating that regional membrane fluidity changes occur during evolution. Putative steroid binding sites overlapping TM-3 occurred in all species. Sites associated with G-protein-receptor stimulation occur both in vertebrates and amphibia but not in *Hydra* or *Paramecia*. The C-terminus moiety “KETYY,” necessary for the Na^+^ activation of pump phosphorylation, is not present in unicellular species indicating the absence of classical Na^+^/K^+^-pumps. The basic protein topology evolved earliest, followed by increases in protein domains and ordered helical arrays, correlated with appearance of α-subunit regions known to involve cell signaling, membrane recycling, and ion channel formation.

## Background

Na/K-ATPase, a highly conserved integral membrane protein, is key to maintenance of both cell volume and electrochemical gradients (Kaplan 2002; Blanco 2005; Geering 2008), as well as cell signaling (Reinhard et al. 2013), has origins that go back to the prokaryotes (reviewed in Saez et al. 2010; Chan et al. 2010). Its α-subunit, responsible for many of its known functions, belongs to a large family of P-type ATPases (reviewed in [Kaplan 2002; Bublitz et al. 2011]). The P-type ATPases, also known as E1–E2 ATPases, are a large group of evolutionarily related ion and lipid transporters that are found in bacteria, archaea, and eukaryotes (Saez et al. 2010). Each consists of bundles of 10 transmembrane (TM) α-helices and is referred to as a P-type ATPase because they catalyze autophosphorylation of a key conserved aspartate residue within the α-subunit. Thever and Saier (2009) analyzed the fully sequenced genomes of 26 eukaryotes for P-type ATPases and reported probable topologies and conserved motifs of 9 functionally characterized families and 13 uncharacterized families of these transporters. Studies by Saez et al. (2010) indicate that the likely origin of many of the P-type (subfamily IIC) proteins is prokaryotic, and that many are present in non-metazoans, such as algae, protozoans, or fungi. They also propose that early deuterostomes presented a single IIC gene, from which all the extant diversity of vertebrate IIC proteins originated by gene and genome duplications.

In this study, we compare the differences in TM helix topology, sequence homology, helix–helix contacts, cell signaling, disordered protein regions, and protein domains of Na/K-ATPase α-subunits in several organisms: (1) *Beauveria bassiana, a fungus*, (2) *Paramecium tetraurelia*, a unicellular ciliate protozoa, (3) *Hydra vulgaris*, a primitive multicellular organism with two main body layers separated by a gel, and (4) both lower (*Xenopus laevis*) and higher vertebrates (*Homo sapiens*). Since the P-type ATPases appear to have evolved into multifunctional integral plasma membrane enzymes during evolution from *Paramecium* to *H.sapiens*, it may be possible to correlate sequential structural changes in the primitive Na^+^-pump (Na/K-ATPase) with the appearance of specialized functions (e.g., cellular signaling, membrane recycling) and in turn with specific sequence changes. As indicated, each α-subunit contains about 1000 residues and 10 TM helices. Structure–function analysis indicates that evolution was due in large part to progressive increases in membrane protein domains and helix–helix interactions. Thus the basic protein topology evolved very early, followed by selective amino acid sequence changes over millions of years that resulted in a complex system that controls cell division, growth, and differentiation.

## Methods

### Protein Sequence Sources

The amino acid sequences of the steroid binding proteins were downloaded from the ExPASy Proteomic Server of the Swiss Institute of Bioinformatics (http://www.expasy.org; http://www.uniprot.org). About 98 % of the protein sequences provided by UniProtKB are derived from the translation of the coding sequences (CDS) which have been submitted to the public nucleic acid databases, the EMBL-Bank/Genbank/DDBJ databases (INSDC). The present study uses the complete amino acid sequences of the α-subunits of the Na/K-ATPase from *B. bassiana* (BEAB2, Accession #J5JMV7), *Paramecium tetraurelia* (ATP1A_PARTE, Accession #Q6BGF7), *H. vulgaris* (AT1A_HYDVU, Accession #P35317), *X. laevis* (AT1A1_XENLA, Accession #Q92123), and *H. sapiens* (AT1A1_HUMAN, Accession #P05023).

### Secondary Structure Predictions

TM helices were predicted using (1) MEMSAT-SVM (Nugent and Jones 2009), (http://www.bioinf.cs.ucl.ac.uk/psipred/), (2) TMpred (Krogh et al. 2001), (http://www.ch.embnet.org/software/tmbase/TMBASF.doc,html), (3) SPOCTOPUS algorithm (Viklund et al. 2008) (http://octopus.cbr.su.se), and (4) MemBrain, which integrates a number of recent bioinformatic approaches including the optimized evidence-theoretic K-nearest neighbor algorithm (Shen and Chou 2008) available at http://chou.med.harvard.edu/bioinf/MemBrain/. Pore-lining regions in TM protein sequences were predicted using the method of Nugent and Jones (2012).

The contribution of intrinsic disorder to protein function and identification of functional sites in disordered regions was estimated by the method of Cozzetto and Jones (2013). Protein domain boundary prediction was estimated using the DomPred server (Bryson et al. 2007) and the Membrain server (Yang et al. 2013). The methods of Bryson et al. (2007) are available at http://bioinf.cs.ucl.ac.uk/software.html. The method of Yang et al. is available at http://www.csbio.sjtu.edu.cn/bioinf/MemBrain .

### TMKink: A Method to Predict Transmembrane Helix Kinks

Meruelo et al. (2011) have identified distinct residue preferences in kinked versus non-kinked helices and have exploited these differences and residue conservation to predict kinked helices using a neural network. The kink predictor, TMKink, is available at http://tmkinkpredictor.mbi.ucla.edu/.

### Helical Packing Arrangement Predictions

The MEMPACK prediction server (psipred@cs.ucl.as.uk) was used to predict lipid exposure, residue contacts, helix–helix interactions, and helical packing arrangement, in addition to TM topology. The MemBrain method (http://csbio.sjto.edu.cn/bioinf/MemBrain/) was used to derive TM inter-helix contacts from amino acid sequences by combining correlated mutations and multiple machine-learning classifiers (Yang et al. 2013). The TOPCONS web interface (http://topcons.cbr.su.se/pred/result/rst_j7ZjE1) allows for constraining parts of the protein sequence to a known inside/outside location to be displayed both graphically and in text format (Bernsal et al. 2009).

## Results and Discussion

### Comparison of Transmembrane Topology of Na/K-ATPase α-Subunits in *Paramecia*, *Hydravulgaris,* and *Homo sapiens*

**Fig. 1:**
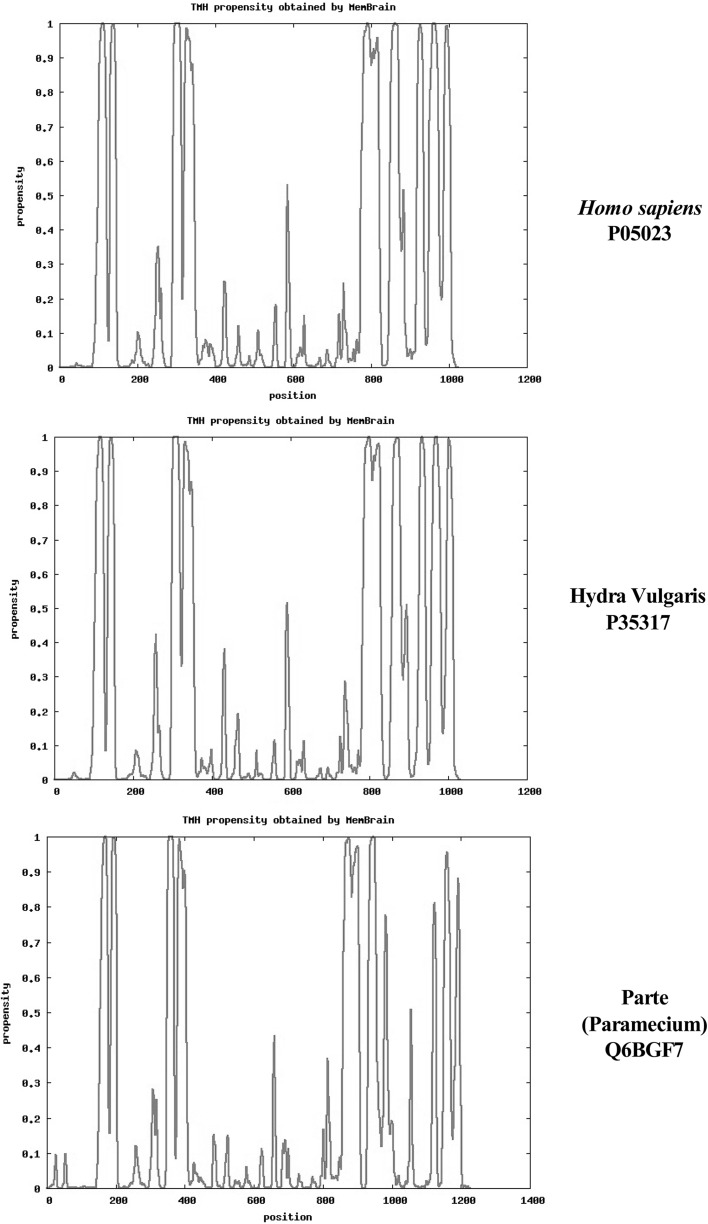
A comparison of the topology of TM helices of Na/K-ATPase α-subunits of *Homo* *sapiens* (*top*, #P05023), *Hydra vulgaris* (*middle*, #P35317), and *Paramecium tetraurelia* (*bottom*, Q6BGF7) using the MemBrain algorithm. The abscissa represents the sequence positions; the ordinate indicates the propensity of TM helices. Plots were obtained as described in “Methods” section

Figure 1 compares the predicted topology of the α-subunits of Na/K-ATPase from *H. sapiens* (top), ATP1A1, Accession #P05023, with a primitive multicellular organism, *H. vulgaris,* (middle) *A*T1A_HYDVU, Accession #P35317 and a unicellular protozoa, *Parameciumtetraurelia*, (bottom) AT1A_PARTE, Accession Q6BGF7, using the MemBrain Server (see Methods” section). The amino acid sequences are those for the complete proteins as published in the Swiss Protein Database (www.uniprot.org). The ordinate indicates the propensity of multiple respective helices using the MemBrain algorithm. In all three species both the N- and C-terminal ends of the α-subunit are cytoplasmic. All exhibit two helix pairs in the N-terminal region and a second group of 6 helices in the C-terminal region. MemBrain is a machine-learning-based predictor (see Methods), which integrates of number of bioinformatics approaches, including sequence representation by multiple sequence alignment matrix, the optimized evidence-theoretic K-nearest neighbor prediction algorithm, fusion of multiple prediction window sizes, and classification by dynamic threshold. MemBrain demonstrates an improvement of about 20 % accuracy in predicting the ends of TM helices, particularly for TM helices shorter than 15 residues. As noted by Shen and Chou (2008), although a significant per cent of the TM helices of known structures are either very short (<15 residues) or very long (>40 residues), prediction methods such as TMHMM (Krogh et al. 2001) cannot detect TM helices shorter than 16 residues or longer than 35 residues. Other algorithms [e.g., TOPCONS (2009)] assume all TM helices contain 20 residues.

As shown in Fig. 1, the MemBrain algorithm predicts 10 TM helices in *H. sapiens* and *Hydravulgaris* but only 9 TM helices plus 2 “half helix” TMs in *Paramecia* (PARTE). For comparison, other servers [e.g., SPOCTOPUS, (Viklund et al. 2008) MEMSAT-SVM, (Nugent and Jones 2012) and Phobius, (Kall et al. 2007)] predict that each species contains 10 TM helices. As can be seen, there is more variability in the C-terminal region, with apparent shifts in position of TM helices 5, 6, and 7 during the evolution of early multicellular organisms (i.e., *Paramecium* to *Hydra*). A similar MemBrain analysis of the topology of other mammalian P-type IIC enzymes Ca-ATPase (SERCA), H-ATPase, H/K-ATPase, and phospholipid flippase predicted that all contain 10 TM helices, with two pairs of TM helices in the N-terminal region, and a group of 6 helices at the C-terminal end (data not shown).

Amino acid sequences of the three proteins shown in Fig. 1 as well as the α-subunit of the Na/K-ATPase a lower vertebrate (*Xenopus laevis*) were compared using the Pairwise Sequence Alignment software (LALIGN) at http://www.ebi.ac.uk/Tools/services/web lalign to detect internal duplications by calculating non-intersecting local alignments (Huang and Miller 1991). Table 1 compares the  % identities of amino acid residues in the 10 TM helices of *X. laevis* (column 2), *H. vulgaris* (column 3), and *P. tetraurelia* (column 4) with those of *H. sapiens* (numbers in parenthesis indicate the  % amino acid similarities). There is a high correlation between *H.sapiens* and *X. laevis*. Comparison of *H. sapiens* and *H. vulgaris* also demonstrated a high correlation between the two TM helical pairs in the N-terminal region; less so within the C-terminal TM helices. On average, *P. tetraurelia* exhibited far fewer sequence correlations and displayed only 10–30 % identity with *H. sapiens* in the 4 C-terminal TM helices.

**Table 1:** Similarities between helices of the α-subunit of Na/K-ATPase in *Xenopus laevis*, *Hydra vulgaris*, and *Paramecium tetraurelia* compared to *Homo sapiens*

Transmembrane (TM) helices	% Identity of amino acid sequences of individual transmembrane helices (% Amino acid similarities indicated in parenthesis)		
	*Xenopus laevis*	*Hydra vulgaris*	*Paramecium tetraurelia*
TM-1	95	90	55
TM-2	100	90	40 (85)
TM-3	95	90	45 (65)
TM-4	100	100	75 (90)
TM-5	100	70 (85)	45 (80)
TM-6	100	100	50 (65)
TM-7	100	80 (85)	30 (45)
TM-8	85 (95)	75	10 (30)
TM-9	100	75	25 (70)
TM-10	85 (100)	65	10 (15)

The Emboss Water protocol (version 36.3.5e Nov, 2012; preload8) used here employs the Smith–Waterman algorithm (with modified enhancements) to estimate the local alignment of two sequences (Huang and Miller 1991). Comparison of *H.sapiens* and *H. vulgaris* α-subunits revealed a Waterman–Eggert score of 4825 with 70.9 % identity (88.5 % similar) in 1020 amino acid overlap (8-1023:14-1031). Comparison of *H. sapiens* and *Paramecium* α-subunits indicated a Waterman–Eggert score of 2404 with 46.6 % identity (74.0 % similar) in 882 amino acid overlap (40-886:99-969).

### Putative Domain Boundaries in Human, Hydra, and Paramecium α-Subunits

The shortest sequence of amino acids in proteins that contains functional and structural information is termed a “motif,” whereas a conserved part of a given protein that can evolve, function, and exist independently is termed a “domain.” Domains form the structural basis of the physiological functions of proteins and each domain can be considered as a semi-independent structural unit of a protein capable of folding independently (Wetlaufer 1973; Richardson 1981; Vogel et al. 2004). A variety of different methodologies have been employed to predict domains but many are fraught with problems since domain assignment is difficult even when the structures are known. Bryson et al. (e.g., 2007) have developed a useful method for computer-assisted protein domain boundary prediction, using the DomPred server (see Methods). This server uses the results from two completely different categories (DPS and DomSSEA). Each is individually compared against one of the latest domain prediction benchmarks to determine their respective reliabilities.

**Fig. 2:**
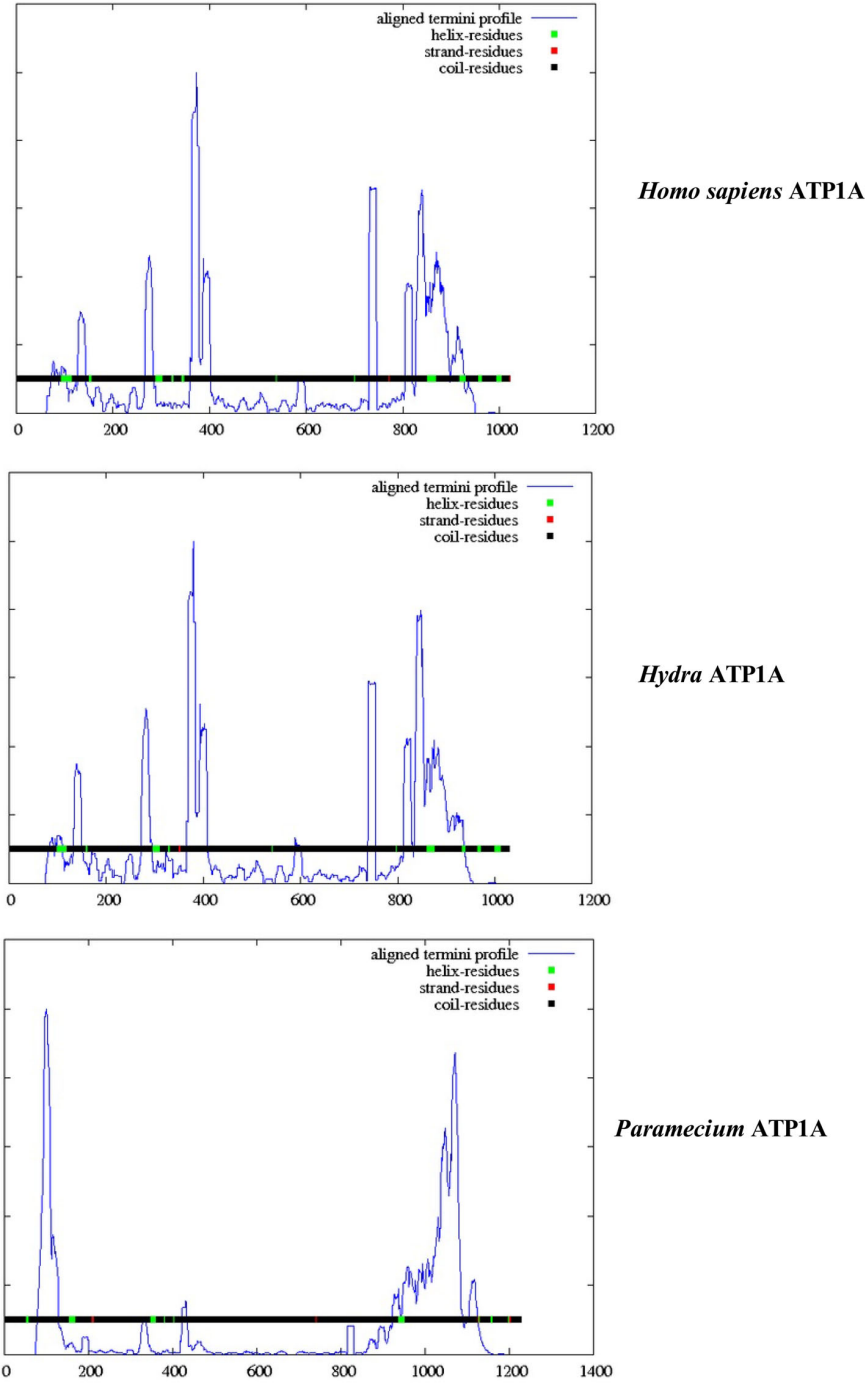
A comparison of the domain topology of the Na/K-ATPase α-subunits from *Homo* *sapiens* (*top*), *Hydra vulgaris* (*middle*), and *Paramecium tetraurelia* (PARTE, *bottom*) using the DomPred server (see “Methods” section). The *vertical peaks* and *bars* indicate the positions of domains within the peptide sequence. The ordinate represents the aligned termini profile and predicts the probability of the respective domains based on the DomPred algorithm

Figure 2 compares the domain topology of the α-subunit of the Na/K-ATPase from *H. sapiens* (top), *H. vulgaris* (middle), and *P. tetraurelia* (bottom), using the DomPred server. Vertical peaks and bars indicate the positions of domains within the peptide sequence. The ordinate represents the aligned termini profile and predicts the probability of the respective domains based on the DomPred algorithm. As shown, the DomPred profiles of *H. sapiens* and *H. vulgaris* are largely identical and more complex than the aligned termini profile shown for *P. tetraurelia*. Compared with *Hydra*, *Paramecium* lacks domains in the 200–800 residue range. The DomPred profile of *Paramecium* indicates that the predominance of domains occurs in the region containing TM helices five through eight (see Fig. 1). This suggests that significant differences in helix–helix interactions may occur in this region of the α-subunit.

### Evolution of Na/K-ATPase as a Signal Transducer

Xie and Askari (2002) were among the first to show that, in addition to pumping ions, Na/K-ATPase interacts with neighboring proteins in cardiac cells to release cascades of signaling proteins which send messages to other intracellular sites. They concluded that exposure of intact cardiac cells to cardiotonic steroids such as ouabain activates growth-related cell pathways by binding to a pre-existing Src-Na/K-ATPase complex. Though the existence of direct contact between Src and Na/K-ATPase has been seriously questioned (Reinhard et al. 2013; Weigand et al. 2012; Gable et al. 2014), the role of Na/K-ATPase as a signal transducer is now well established (Reinhard et al. 2013). To date, two ouabain-activated pathways have been identified: the EGFR/Src/Ras/ERK pathway and the P13 K1A-PDK-Akt pathway, both of which affect growth in a variety of cell types (Wu et al. 2013). Another signaling pathway involving Na/K-ATPase is that reported by Yudowski et al. (2000), resulting in the endocytosis of Na/K-ATPase molecules in response to G-protein-coupled receptor stimulation of the PI3K-IA/PDK/Akt pathway. As discussed by Wu et al. (2013) and Yudowski et al. (2000), it is most likely that the interaction of the Na/K-ATPase with PI3K1A is direct and through the binding of the SH3 domains of the p-85 subunit of PI3K1A to the proline-rich domain (TPPPTTP) of the α-subunit of Na/K-ATPase. As shown in Table 2 (column 2), the proline-rich domain is not common in species prior to Hydra. This is consistent with the finding by Mushegian et al. (2012) that G-protein-coupled receptors apparently emerged before vertebrates and rapidly expanded in true Metazoa, probably due to the need for rapid signaling adjustments in fast-moving species.

**Table 2:** Comparison of different regulatory systems associated with cell signaling in the Na/K-ATPase α-subunit

Na/K-ATPase α-subunit	Activation of phosphatidylinositide 3-Kinase 1A/Akt	Putative progesterone cell surface binding site first external loop	C-terminal contacts that stabilize Na-pump conformations
*Homo sapiens* P05023	^81^TPPPTTP^87^	^118^QAATEEEPQN^127^	KETYY
*Xenopus laevis Q92123*	^83^TPPPTTP^89^	^119^QAAMEEEPQN^131^	KESYY
*Hydra vulgaris* P35317	^87^TPPKQTP^93^	^125^AVRDTNPNM^133^	KETYY
*Paramecium* Q6BGF7	^78^APTNTKQ^84^	^177^VNPEALGAKS^184^	ANSLW
*Beauveria bassiana* J5JMV7	^69^NPLSWVM^75^	^171^KVLGYVFGGFCSVLW^184^	AKIAW
*Vicia faba* Q43131	^68^PSSPYTGI^75^	^86^ANGGGQPPDWQ^96^	YKGNT

Studies using isolated plasma-vitelline membranes from *Rana pipiens* oocytes have shown that progesterone may act as a meiotic agonist by binding to the external loop sequence (QAATEEEPQN) between TM-1 and TM-2 helices of the α-subunit (Morrill et al. 2008) and rapidly activate lipid enzymes such as *N*-methyl transferase and sphingomyelin synthase (Morrill et al. 2010). As shown in Table 2, both *H. sapiens* and *X. laevis* contain the putative progesterone binding site, whereas *Hydra vulgaris* and lower forms do not. Voltage-clamp measurements by Vedovato and Gadsby (2010), using intact *Xenopuslaevis* oocytes indicate that the two C-terminal tyrosines (YY) of the α-subunit stabilize Na/K-pump conformations. Deletion of the last five residues (KETYY) of the α-subunit markedly lowers the apparent affinity for Na^+^ activation of pump phosphorylation (Vedovato and Gadsby 2010). Table 2 further indicates that humans, frogs, and hydra all contain the C-terminal KET(or S)YY contacts, whereas paramecia and the plasma membrane ion transporter ATPase of plant (*Vicia faba*) cells do not. This indicates that both the P13K1A-PDK-Akt pathway and the C-terminal YY residues play important roles in primitive multicellular species such as *H. vulgaris* (column 2), but not in lower species. Table 2 also indicates that progesterone binding does not contribute to *H. vulgaris* physiology, suggesting that a steroid response system(s) arose at a later stage of evolution.

### Predicting Helix–Helix Interactions in the α-Subunits Based on Residue Contacts

**Fig. 3:**
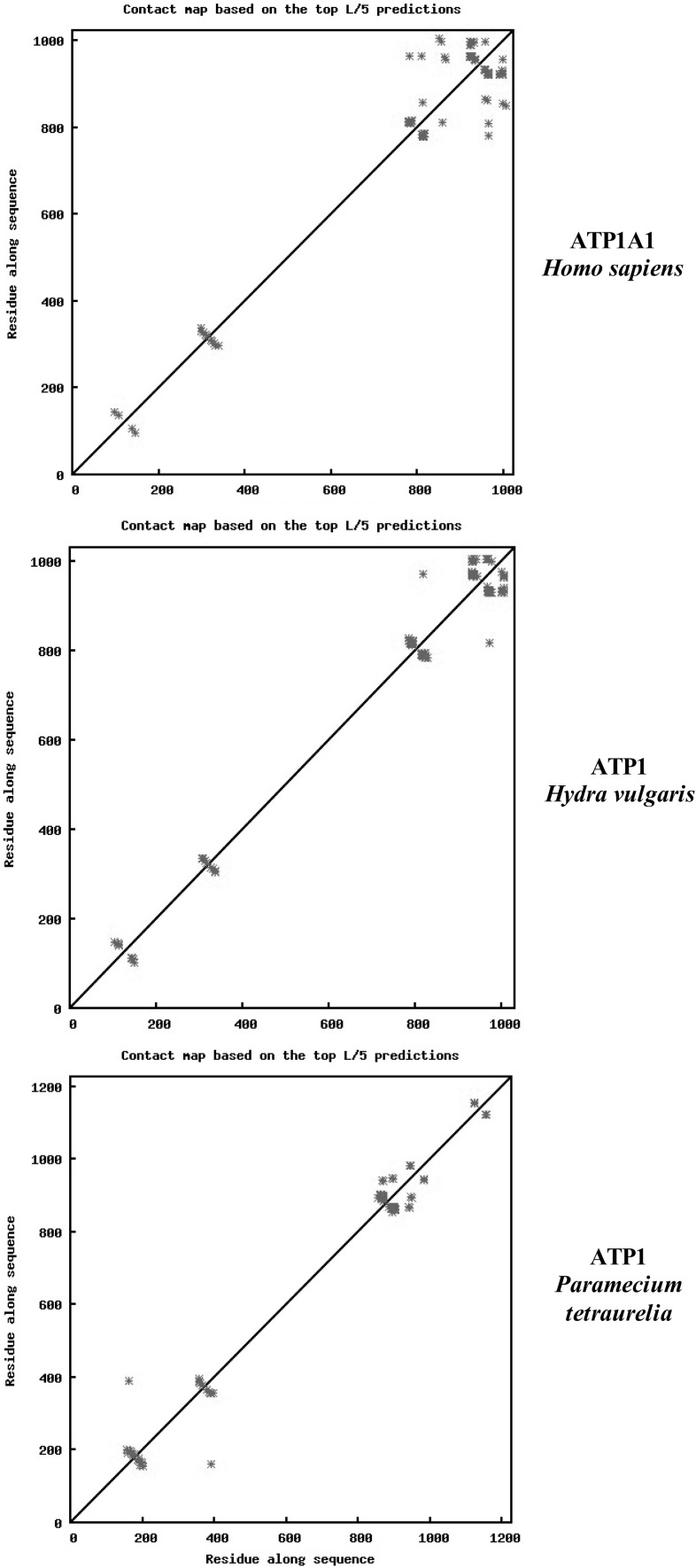
Comparisons of the contact maps based on the top L/5 predictions for ATP1A1 of *Homo sapiens* (*top*), *Hydra vulgaris* (*middle*), and *Paramecium tetraurelia* (*bottom*). Plots were obtained as described in “Methods” section

Residue–residue contacts within the TM helices determine the three-dimensional topology of the α-helical membrane proteins. Yang et al. (2013) have developed a method using the PSICOV algorithm to calculate correlated mutation scores. The ML-based engine was in turn merged with the CMA-based approach. Figure 3 illustrates the contact maps based on the top L/5 predictions for ATP1A1 of *H. sapiens* (top), *H. vulgaris* (middle), and *P. tetraurelia* (bottom). Contacts occurred in the 100 and 400 residue regions in all three species. Contacts increased markedly in the C-terminal region going from *Paramecium* to *Hydra* to *H. sapiens*. In contrast, contacts decreased in the 900 residue region when Hydra was compared to *Paramecium*.

**Fig. 4:**
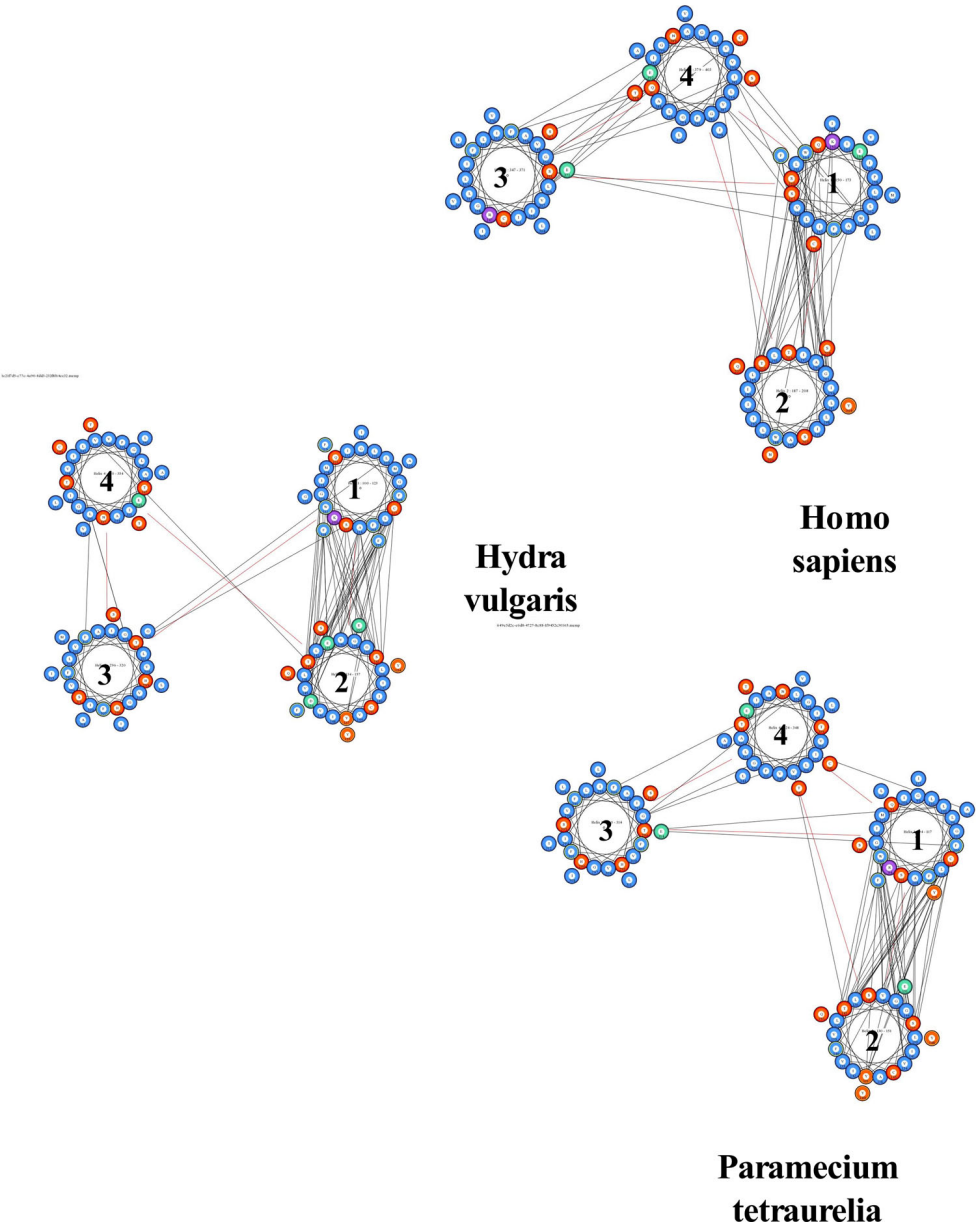
A comparison of four MEMPACK-SVM defined TM helices (TM-1–TM-4) in the N-terminal regions of α-subunits of *Homo sapiens* (*top graphic*, P05023), *Hydra vulgaris* (*middle graphic*, P35317), and *Paramecium tetraurelia* (*bottom graphic*, Q6BGF7). *Colors* in the MEMPACK cartoon indicate hydrophobic residues (*blue*), polar residues (*red*), and charged residues (*green* for negative, *purple* for positive). *Lines* between residues indicate predicted helix–helix interactions

An approach developed by Nugent and Jones (2009, 2012) using the MEMPACK alpha-helical TM protein structure prediction server (http://bioinf.cs.ucl.ac.uk) allows comparison of the interactions between specific helices. Figure 4 illustrates helix–helix interactions between the first 4 helices in *H. sapiens* (top right), *H. vulgaris* (middle left), and *Parameciumtetraurelia* (bottom right). The cartoon illustrates the MEMSAT-SVM helix orientation and the predicted packing arrangement of the N-terminal region. Colors in the MEMPACK cartoon indicate hydrophobic residues (blue), polar residues (red), and charged residues (green for negative, purple for positive). Lines between residues indicate a predicted interaction. As can be seen, all three species demonstrate significant interaction between TM-1 and TM-2. A further increase in helix–helix interactions between TM-1, TM-3, and TM-4 occurs in *H. sapiens*.

**Fig. 5:**
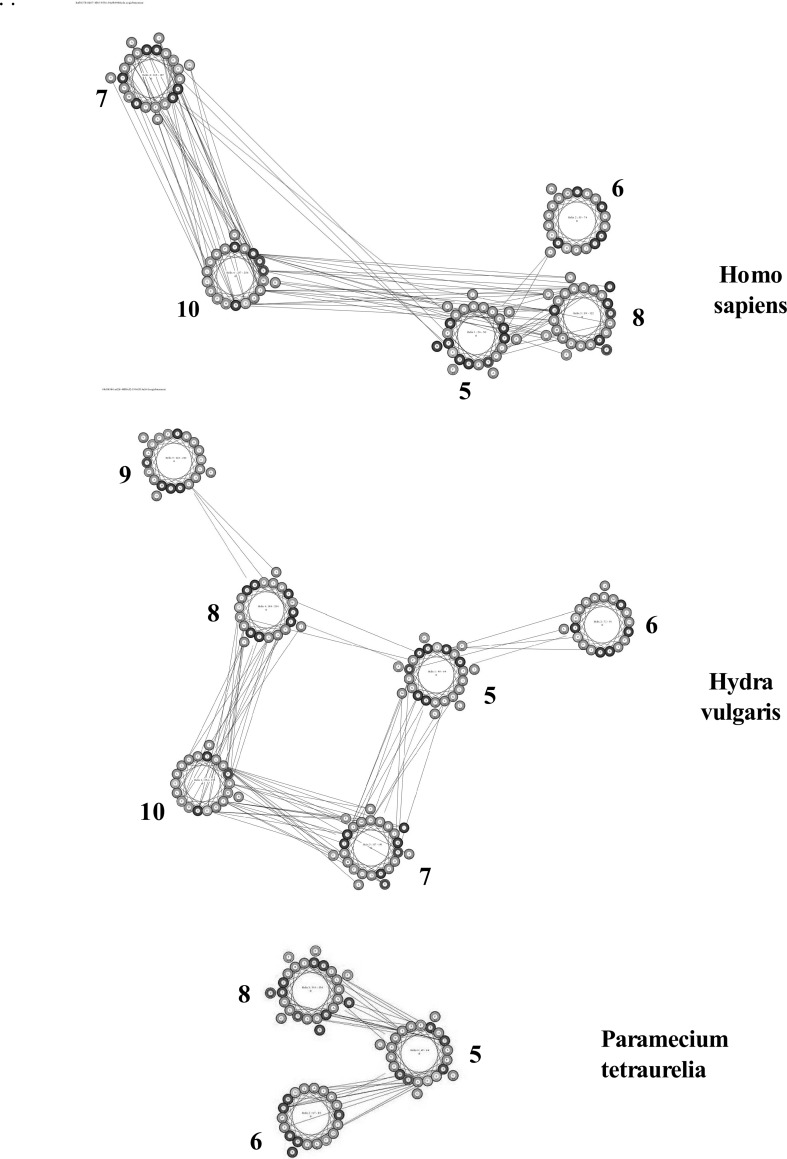
A comparison of six MEMPACK-SVM defined TM helices (TM-5–TM-10) in the C-terminal region of α-subunits of *Homo sapiens* (*top graphic*, P05023), *Hydra vulgaris* (*middle graphic*, P35317), *Paramecium tetraurelia* (*bottom graphic*, Q6BGF7). Predicted TM helices are indicated in *black*. *Blue squares* indicate predicted pore-lining regions. Extracellular regions (membrane loops) are in *orange*. See “Methods” section for details

A comparison of the TM helix packing arrangements of the 6 C-terminal TM helices in *Paramecium*, *Hydra*, and *H. sapiens* indicates increasing TM helix–helix interactions in the α-subunit of Na/K-ATPase, going from paramecium to humans (Fig. 5). Interactions between the six C-terminal helices of the domain-rich region of *Paramecium* are restricted to interactions between TM-5, TM-6, and TM-8. By comparison, *Hydra* demonstrates helix–helix interactions within all six helices with maximal interactions between TM-10 and both TM-7 and TM-8. *H. sapiens* demonstrate a similar extensive helix–helix interaction between TM-10 and TM-7 and TM-8, but no longer interact with TM-9.

### Comparison of Pore-Lining Regions, Hydrophobic Cores, and Disordered Regions in α-Subunits of Several Species

**Fig. 6:**
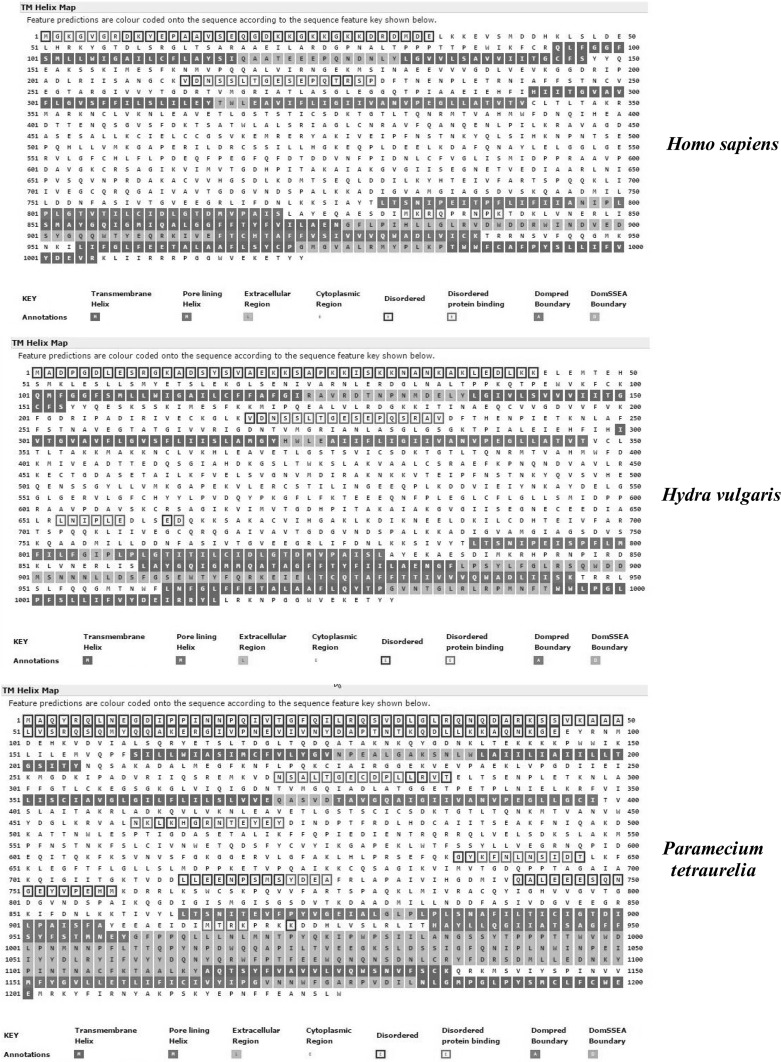
Comparisons of the topology of TM helices, disordered regions, and pore-lining regions of Na/K-ATPase α-subunit of *Homo* *sapiens* (*upper plot*), *Hydra vulgaris* (*middle plot*), and *Paramecium* *tetraurelia* (*lower plot*). *Blue squares* indicate predicted pore-lining regions and *black squares*, non-pore helix regions. Predicted TM helices are indicated in *black*. *Blue squares* indicate predicted pore-lining regions. Extracellular regions (membrane loops) are in *orange*. Disordered regions are indicated as open boxes outlined in either *red* (disordered) or *green* (Disordered protein binding). See “Methods” section for details

As noted in the Introduction, the protein structure of the α-subunit of Na/K-ATPase is crucial in facilitating the passage of ions via channels across lipid bilayers. Typically, channel proteins contain a cavity (or pore) which spans the entire molecule with an opening on each side of the membrane. The pore often runs parallel to TM helices, forming the path along which ions travel, with adjacent compositional or structural features determining pore specificity (reviewed in Nugent et al. 2011). Nugent and Jones have developed methods to identify pore-lining regions in TM proteins from sequence information alone, which can then be used to determine pore stoichiometry (Nugent 2015). In addition, recent developments which identify functional sites in disordered regions can provide insight into their biochemical and cellular functions (Benito et al. 2002). Figure 6 compares the TM helix maps for *H. sapiens* (top), *H. vulgaris* (middle), and *Parameciumtetraurelia* (bottom). As defined in the annotations (bottom), predictions for TM helices are indicated as black squares, pore-lining regions as blue squares, intracellular sequences are white, and extracellular sequences are orange. The disordered regions are identified by open boxes outlined in red (disordered) or green (disordered protein binding). As shown in Fig. 6, sequences previously identified as TM helices (TM-4, TM-5, TM-6, and TM-8) are identified as pore-lining regions in *H. sapiens* and *Hydra*. *H. sapiens* have several pore-lining regions in common with *Hydravulgaris* (TM-4, TM-5, TM-6, TM-7 and TM-9). The major difference in topology occurs in the C-terminal region of *P. tetraurelia*. Figure 6 also indicates that the disordered regions are located in the cytoplasmic regions (open boxes with colored outlines in Fig. 6) and that disorder largely disappears between Paramecium and Hydra, with a subsequent disappearance of disorder within the large intracellular loop between TM-4 and TM-5 as well as the N-terminal region.

**Fig. 7:**
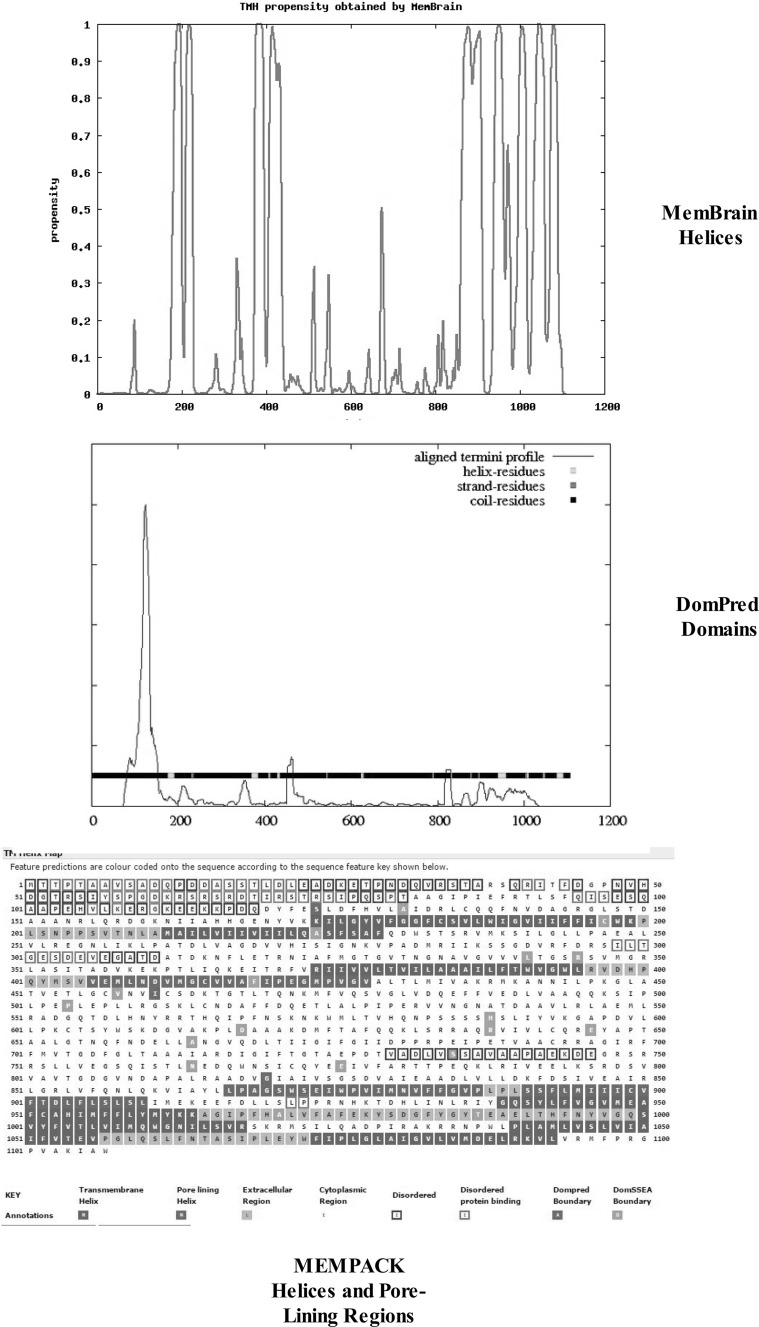
Comparisons of the topology of TM helices (*top graphic*), protein domains (*middle graphic*), and disordered and pore-lining regions (*lower graphic*) of the Na/K-ATPase α-subunit of *Beauveria bassiana* (strain ARSEF 2860, White Muscardine disease fungus, #J5JMV7). *Blue squares* indicate predicted pore-lining regions and *black squares*, non-pore helix regions. Predicted TM helices are indicated in *black*. *Blue squares* indicate predicted pore-lining regions. Extracellular regions (membrane loops) are in *orange*. Disordered regions are indicated as *open boxes* outlined in either *red* (Disordered) or *green* (Disordered protein binding). See Methods for details

K^+^- or Na^+^-efflux ATPases are also key enzymes in the evolution of fungi (Benito et al. 2002). In fungi, a Na^+^/cation exchange system has evolved to compensate for elevated internal concentrations of Na^+^ (reviewed in Benito et al. 2002). As shown in Fig. 7, analysis of the Na^+^/K^+^ ATPase alpha 1 subunit of the fungus *B. bassiana* (J5JMV7) indicates that the α-subunit has 10 TM helices with the familiar pattern of two pairs in the N-terminal region and a cluster of 6 helices in the C-terminal region (top graphic). MEMPACK analysis (bottom graphic) further indicates TM-4, TM-5, TM-6, and TM-8 are pore-lining regions in fungus. In comparison, TM-4, TM-5, TM-6, and TM-9 are pore-lining regions in *Paramecium* (Fig. 6)*. Hydra* and *H. sapiens* differ in that TM-8, not TM-9, is a pore-lining region. The domain topology of the fungus *B. bassiana* (middle graphic of Fig. 7) is much simpler than that seen for *Paramecium*, *Hydra*, or *H. sapiens* (Fig. 2). In *Beauveriabassiana*, identifiable protein domains are limited to residues 50–150.

It should be noted that about half of all TM helices are found to contain bends and other deviations, often referred to as “kinks” (Hall et al. 2009; Langelaan et al. 2010). As shown by Meruelo et al. (2011), distortions in helix geometry may facilitate conformational changes required for protein function by providing sites of flexibility (Bright et al. 2002; Shi et al. 2002) and can be important for positioning key residues precisely in the protein structure. Kinks that open the polar backbone to alternative hydrogen bonds often attract water molecules, thus providing a polar region within the hydrophobic core. Using TMKink to predict TM helix kinks in the α-subunits, Table 3 indicates which of the ten TM helices (and/or pore-lining regions) contain kinks. As indicated, only two of the ten helices of *Paramecia tetraurelia* (4, 7) contain kinks, whereas eight of the ten helices (1, 2, 3, 4, 7, 8, 9, and 10) of *Hydra* contain kinks. In comparison, all four of *H. sapiens* N-terminal TM helices (1, 2, 3, 4) contain kinks. There appears to be little correlation between kinks and pores. Only pore-lining region 4 contains a kink in all three species.

**Table 3:** Comparison of pore-lining regions and predicted kinks by position in *Paramecia tetraurelia,* *Hydra vulgaris*, and *Homo sapiens*

Transmembrane (TM) helix	*Paramecia tetraurelia*		*Hydra vulgaris*		*Homo sapiens*	
	TM kinks	Pore regions	TM kinks	Pore regions	TM kinks	Pore regions
TM-1			^114^A–I^123^		^139^V–S^147^	
TM-2			^145^V–S^153^		^275S^S–A^285^	
TM-3			^281^S–A^291^		^311^I–A^320^	
TM-4	^335^E–I^343^	Pore	^294^I–I^300^	Pore	^333^V–V^340^	Pore
TM-5		Pore		Pore		Pore
TM-6		Pore		Pore		Pore
TM-7	^794^H–K^810^	Pore	^818^I–S^829^			
TM-8			^872^A–G^893^	Pore		Pore
TM-9		Pore	^910^F–I^922^			
TM-10			^957^M–G^965^			

## Conclusions

The emerging pattern suggests that the catalytic α-subunit of Na/K-ATPase must have evolved as a result of the sequential introduction of protein domains within the C-terminal region of a ~ 1000 residue precursor polypeptide. The α-subunit of a fungus, *B. bassiana* exhibits a similar number of amino acid residues (1107 and 1023) and the same topology as that of *H. sapiens*; e.g., 2 pairs of TM helices in the N-terminal region and a cluster of 6 TM helices in the C-terminal region (Figs. 1, 6, 7). The TM helices and pore-lining regions (Figs. 6, 7) probably evolved early and the topology has remained unchanged over millions of years. A primary difference between *Beauveria*, *Paramecium*, and the early multicellular organism (*H. vulgaris*) is the progressive increase in the number and complexity of the protein domains (Figs. 2, 7).

The membrane potential of most fungal cells is about −150 mV [cf. (Shi et al. 2002)]. This high membrane potential is the driving force that supports the function of many uniporters, symporters, and antiporters that fungi have evolved to balance excessive Na^+^ uptake. The limited distribution of protein domains to the C-terminal region of fungi indicates that the uniporters, symporters, and/or antiporters are largely associated with protein domains within the first 250 residues of the N-terminal region (compare Figs. 2, 7). *Paramecia* lack the classical Na/K-ATPase, since K^+^ does not stimulate Na^+^ efflux (Hansma 1979), consistent with the absence of the C-terminal KETYY sequence (Table 2). In *Paramecium*, ionic currents control swimming behavior (Eckert and Brehm 1979). When its membrane potential is at the resting level, *Paramecium* swims forward. When its membrane is hyperpolarized it swims backward. A closely related ciliate, *Tetrahymena*, also appears to lack a classical pump Na/K-ATPase, since the specific inhibitor, ouabain, has no effect on Na^+^ and K^+^ transport (Dunham and Kropp 1983). The absence of the C-terminal KETYY sequence in both fungi and *Paramecium* (Table 2) is evidence for limited α-subunit functionality in lower species.

As shown in Fig. 2, the catalytic subunit of a primitive multicellular organism, *H. vulgaris,* contains many of the protein domains characteristic of the ATP1A1 α-subunit of *H. sapiens,* indicating that much of Na-pump evolution occurred during the development of multicellular organisms. The ectodermal epithelium of the fresh water *H. vulgaris* is the main site of ionic regulation in these organisms (Chain 1980). It actively transports Na^+^ from the environment into the enteron and extracellular fluids, which are the two milieus that act as these animals’ only “internal environment.” A major step in electrical potential occurs across the inner ectodermal membrane; produced by an inwardly directed electrogenic pump which is sensitive to ethacrynic acid but not to ouabain (Chain 1980). As indicated in Table 2, the C-terminus of the *H. vulgaris α*-subunit contains the moiety “KETYY,” reported to be necessary for the Na^+^ activation of pump phosphorylation from ATP in vertebrates (Vedovato and Gadsby 2010). The lack of “KETYY” in *Paramecium* and *B. bassiana* indicates that neither contains classical Na^+^/K^+^-pumps.

An important observation was that the introduction of multiple protein domains into the α-subunit occurred during the evolution of primitive multicellular organisms (Fig. 2). Most protein domains were introduced within the 300–400 residue region containing TM-3 and TM-4 and to a lesser extent within the 800–900 residue region containing TM-5 and TM-6. As shown in Table 3, this region is associated with a marked increase in TM kinks. As noted above, Meruelo et al. (2011) suggest that TM kinks produce distortions in helix geometry and may be important for positioning key residues precisely within the protein structure, which together with amino acid sequence changes may lead to the formation of new protein domains. Analysis of caveolin-binding motifs (Table 4), known to be associated with membrane lipid rafts and cell signaling (Epand et al. 2005), reveal that significant positional shifts occur between *Paramecium* and *H. vulgaris,* indicating that regional membrane fluidity changes occur during evolution. Lipid rafts display reduced lateral diffusion relative to the lipid-disordered phase, thus providing nucleation sites for the further membrane organization to produce larger structures of 50–150 nm (reviewed in Epand et al. 2005). Table 4 also compares the locations of both caveolin-binding motifs and leucine-rich repeats in the catalytic subunits of Na/K-ATPase in *Paramecium*, *Hydra vulga*ri*s*, and *H. sapiens,* as well as in *X. laevis* (Q92123) and the plant P-type ATP1 (*Vicia faba*, Q43131). Leucine-rich repeats are 20 to 29-residue sequence motifs present in proteins that appear to provide a structural framework for the formation of protein–protein interactions (Kobe and Kejava 2001). As shown for *Paramecium,* caveolin-binding motifs (CBMs) occur between TM-4 and TM-5 and between TM-7 and TM-8, whereas only one CBM occurs in *Hydra* which undergoes an apparent shift to overlap TM-1 (column 2). The two higher species (*Xenopus laevis* and *Homo sapiens*) as well as a higher plant (*Vicia faba*) each contain two CBMs overlapping TM-1 and TM-10. In contrast, each leucine-rich repeat underwent changes in amino acid sequence but remained relatively fixed, overlapping TM-3 in all species.

**Table 4:** Comparison of caveolin-binding motifs and leucine-rich repeat sequences in the α-subunit of various animal and plant cells

Species	Caveolin-binding motif	Leucine-rich repeat
*Homo sapiens* (P05023)	^89^WIKFCRQLFGGFSMLLW^105^ Overlaps TM-1	^308^ILSLILEYTWL^318^
	^987^WWFCAFPYSLLIFVY^100^1 Overlaps TM-10	
*Xenopus laevis* (Q92123)	^91^WVKFCRQLFGGF^102^ Overlaps TM-1	^310^ILSLILQYTWL^320^
	^989^WWFCAFPYSLIIFIY1003 Overlaps TM-10	
*Hydra vulgaris* (P35317)	^95^WVKFCKQMFGGF^106^ Overlaps TM-1	^303^GVAYFLGSFLI^314^
*Paramecium tetraurelia* (Q6BGF7)	^650^FKLEGFTF^657^ Between TM-4 and TM-5	^369^LGILFLILSLVV^370^
	^1052^YYDLRYIFVYYDQNYQRW^1069^ Between TM-7 and TM-8	
*Vicia faba* (Q43131)	^91^WVKFCRQLFGGF^102^ Overlaps TM-1	^338^LNKISVDRNLI^348^
	^989^WWFCAFPYSLIIFIY^1003^ Overlaps TM-10	

The observations presented here indicate that a number of molecular traits of the Na/K-ATPase catalytic subunit became determined by the single-cell stage. These include protein sequence length, TM helix topology, and protein motifs such as the Leucine-rich repeats. Subsequent evolutionary changes appear to be due largely to introduction of protein domains into the catalytic subunits during evolution. A protein domain has been defined as a conserved part of a given protein sequence that can evolve, function, and exist independently (reviewed in Vogel et al. 2004). Each domain forms a compact three-dimensional structure and often can be independently stable and folded to create proteins serving different functions. Domains vary in length from about 25 amino acids to about 500 amino acids in length. Because they are independently stable, they may be exchanged between proteins leading to the evolution of protein families such as the P-type ATPases, which by the end of 2014 had 493 confirmed and unique members in the Swiss-Prot Database (George et al. 2004, Prosite motif PS00154).
